# Recent developments in copper-catalyzed radical alkylations of electron-rich π-systems

**DOI:** 10.3762/bjoc.11.248

**Published:** 2015-11-23

**Authors:** Kirk W Shimkin, Donald A Watson

**Affiliations:** 1 Department of Chemistry and Biochemistry, University of Delaware, Newark, Delaware 19716, United States

**Keywords:** alkene, alkylation, alkyne, catalysis, copper, nitroalkane, radical

## Abstract

Recently, a number of papers have emerged demonstrating copper-catalyzed alkylation reactions of electron-rich small molecules. The processes are generally thought to be related to long established atom-transfer radical reactions. However, unlike classical reactions, these new transformations lead to simple alkylation products. This short review will highlight recent advances in alkylations of nitronate anions, alkenes and alkynes, as well as discuss current mechanistic understanding of these novel reactions.

## Introduction

Atom transfer radical (ATR) reactions are extremely useful in organic synthesis and polymer chemistry [1]. These reactions have been used extensively in polymer chemistry because of the ability of alkyl radicals to participate in chain reactions [2–4]. They have also been used extensively in small molecule synthesis, particularly in an intramolecular cyclization setting [5–9]. However, while the ability of alkyl radicals to add to electron-rich π-systems is well established [10–13], harnessing this mode of reactivity to provide products of intermolecular alkylation would be highly valuable in organic synthesis. Although various metals have been shown to promote radical polymerizations, copper is by far the most common and effective [14].

Recently, several groups, including our own, have developed copper-catalyzed alkylation reactions of molecules containing electron-rich π-systems. These reactions all utilize alkyl halides as the alkylating agent, and deliver reactivity that is not observed in the non-catalyzed reactions. Mechanistically, it is believed that these reactions all share features of classic ATR reactions, but are distinct as they deliver simple alkylation products. These transformations represent a new paradigm in copper-catalyzed radical reactions. This article will highlight recent examples from this emergent area, including copper-catalyzed alkylation reactions of nitroalkanes, alkenes and alkynes.

## Results and Discussion

### Additions to nitronate anions

**Scheme 1:**
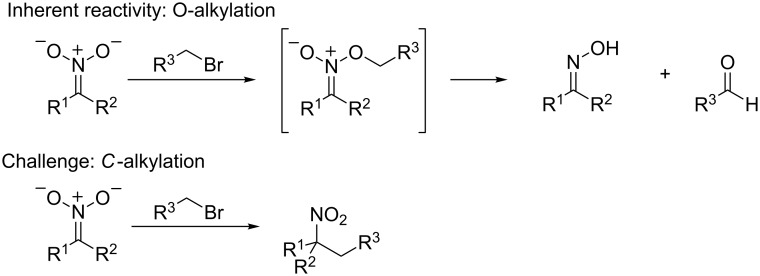
Reactivity of nitronate anions towards alkyl electrophiles.

The selective *C*-alkylation of nitroalkanes with alkyl halide electrophiles is a long-standing challenge in organic synthesis (Scheme 1). This is mainly because of the propensity of nitronate anions to undergo alkylation at oxygen rather than carbon, a process that has been known for more than 60 years [15]. Recently, our group has developed a copper/diketimine catalyst system to selectively *C*-alkylate nitroalkanes [16]. While nitronate anions are known to undergo alkylation with alkyl radicals, these radicals are often accessed using highly undesirable methods such as stoichiometric alkylmetal reagents or highly complex alkylating agents [17]. Although allylation [18–23] and arylation [24–26] of nitroalkanes using palladium catalysis were known, prior to our work, no general catalytic methods for the alkylation of nitroalkanes existed.

**Scheme 2:**
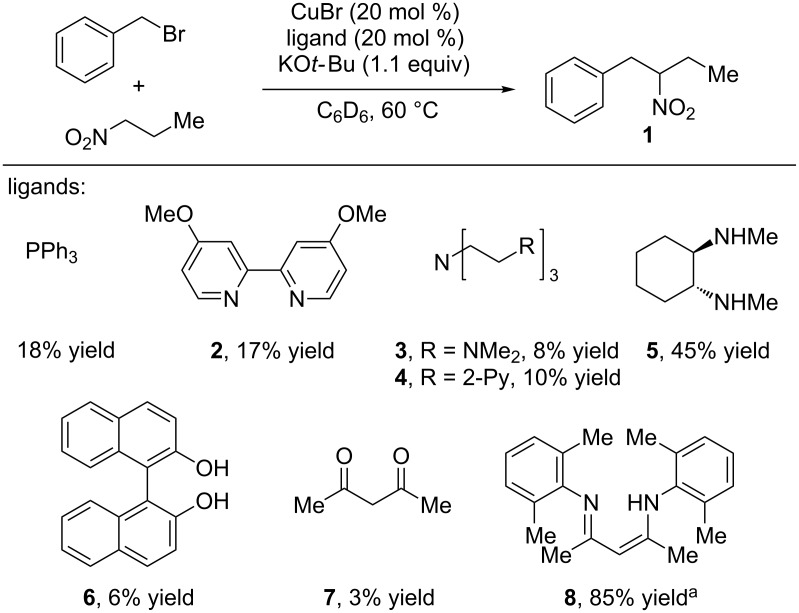
Ligands tested in the alkylation of nitroalkanes with alkyl halides. ^a^NaO*t*-Bu as base, hexanes as solvent.

We envisioned that the catalytic generation of an alkyl radical under copper catalysis in the presence of a nitronate anion could lead to *C*-alkylated products. In optimizing the cross coupling of nitroalkanes and benzyl halides, it was clear that the nature of the ligand had a profound effect on the reaction. Various multi-dentate amine ligands, which are commonly used in ATR reactions, were not effective in the reaction, affording low yields of product and significant amounts of products resulting from *O*-alkylation (Scheme 2). β-Diketimine (nacnac) ligands were determined to be highly effective for the reaction, providing high yields of *C*-alkylated products and only trace amounts of *O*-alkylated products. These types of ligands have recently found use in copper catalyzed C–H amination reactions, as reported by Warren and co-workers [27–28].

**Scheme 3:**
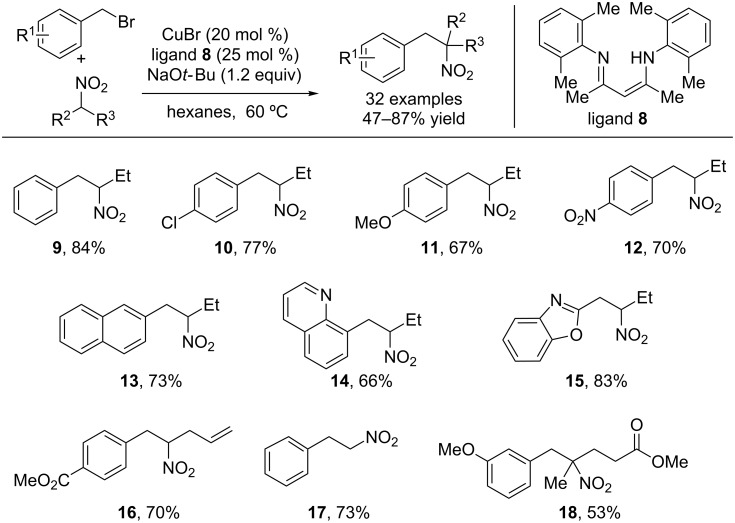
Scope of the copper-catalyzed nitroalkane benzylation.

**Scheme 4:**
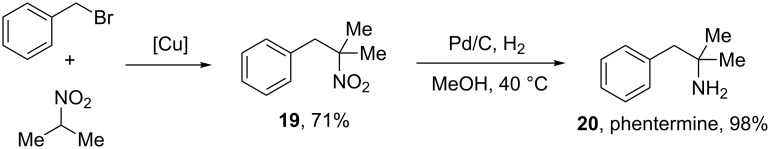
Application of the nitro-alkylation reaction to the synthesis of phentermine.

Using this mild copper-catalyzed system, β-arylnitroalkanes were produced in excellent yields (Scheme 3) [16]. The scope of this transformation is remarkably broad with respect to both coupling partners. Electron-rich, electron-poor and heterocyclic bromides were tolerated in very good yields. Primary and secondary nitroalkanes as well as nitromethane could be cross-coupled in excellent yields, providing access to nitroalkanes with diverse substitution patterns. Additionally, reduction of these products provides phenethylamine derivatives, many of which possess known biological activity. For instance, reduction of the fully substituted nitroalkane **19** provides phentermine (**20**), a clinically prescribed appetite suppressant (Scheme 4) [29–30].

**Scheme 5:**
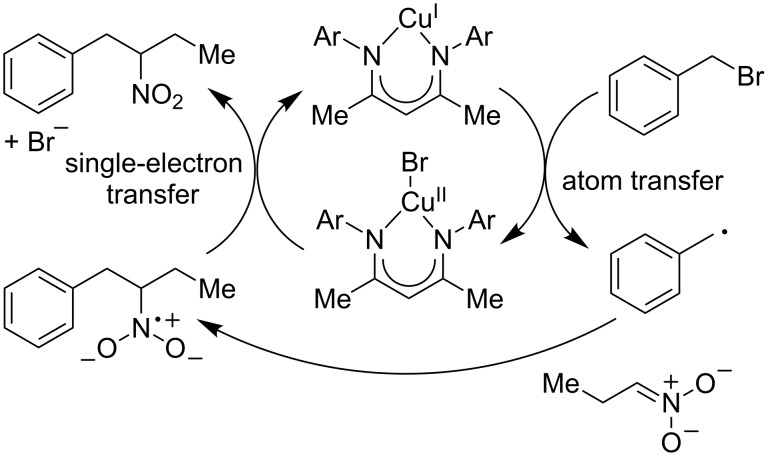
Possible mechanism of the thermal redox process.

While the precise mechanism of this transformation is not fully understood, evidence suggests that radical intermediates are involved in the bond forming step (Scheme 5). For instance, bibenzyl byproducts were observed in the cross-coupling of benzyl halides, suggesting a homodimerization of benzylic radicals. The reaction does not proceed in the presence of radical scavengers such as TEMPO. Radical clock experiments provided ring-opened products, suggesting the presence of intermediate radicals. We propose that this reaction proceeds via a thermal redox process. We hypothesize that the alkyl radical is formed by transfer of a bromine atom from the alkyl halide to the copper catalyst. The resultant stabilized alkyl radical then undergoes coupling with a nitronate anion, forging the C–C bond. Single electron transfer from the resultant radical anion to the Cu(II) halide results in the observed product while simultaneously reducing the metal center to regenerate the catalyst.

**Scheme 6:**
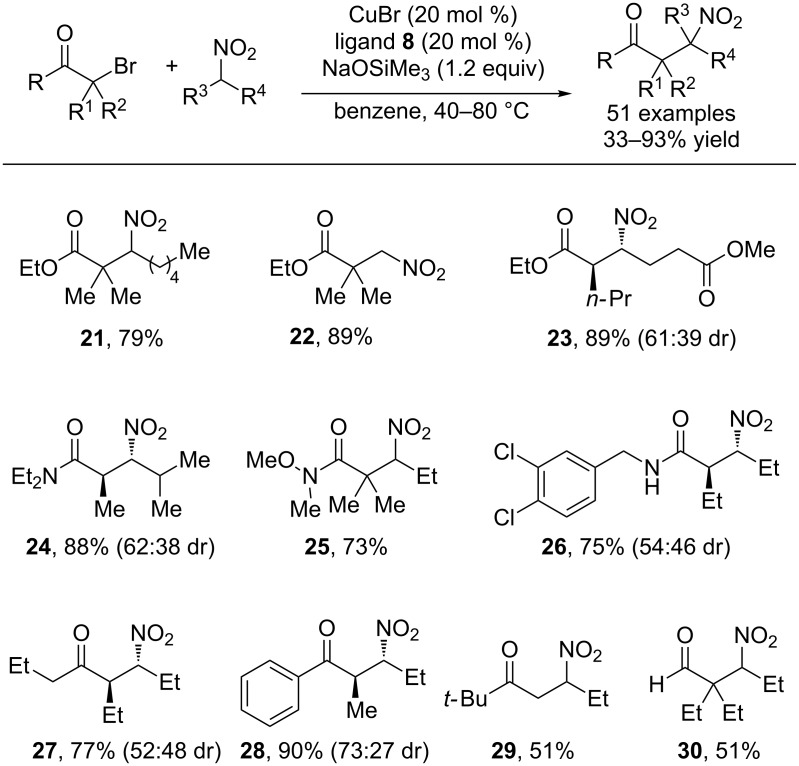
Scope of the reaction of nitroalkanes with α-bromocarbonyls.

**Scheme 7:**
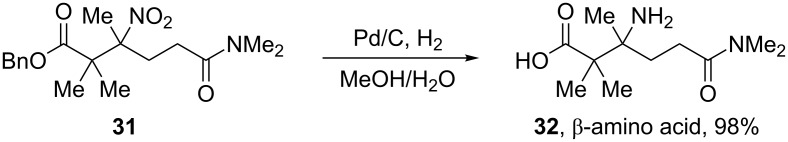
Synthesis of highly congested β-amino acids.

In subsequent studies, α-bromocarbonyls were shown to be effective coupling partners for nitronate anions under similar conditions (Scheme 6) [31]. Remarkably, α-bromo esters, ketones, amides and aldehydes all coupled efficiently with nitroalkanes to provide β-nitrocarbonyls in excellent yields. The steric threshold of this reaction is also highly notable in that β-nitrocarbonyls bearing contiguous quaternary carbons could be synthesized. Because of the ease of reduction of the nitro group, this cross-coupling procedure provides facile access to highly substituted β-amino acids (Scheme 7).

### Additions to alkenes and alkynes

**Scheme 8:**
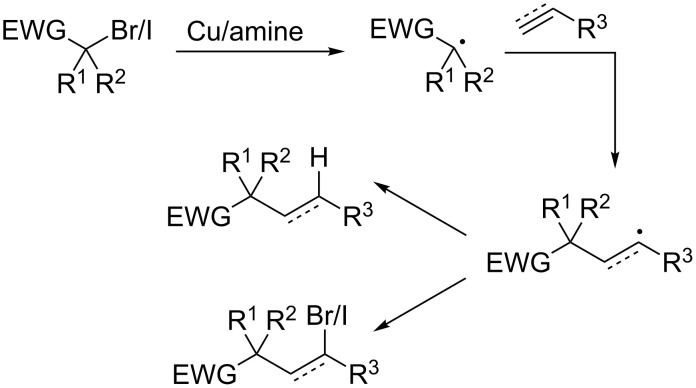
Copper-catalyzed alkenylation reactions.

One of the burgeoning areas of copper-catalyzed cross-coupling chemistry is the alkenylation of alkyl halide substrates. This transformation is achieved via formation of a transient radical, addition to a π-system (an alkene or an alkyne), followed by elimination or reincorporation of a halide atom. This general scheme is outlined in Scheme 8.

**Scheme 9:**
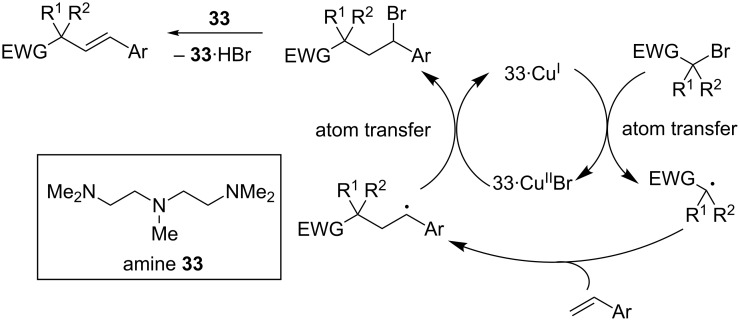
Proposed mechanism of the copper-catalyzed alkenylation reaction.

In 2013, Nishikata and co-workers reported that a copper/amine catalyst system catalyzes the alkenylation of tertiary alkyl bromides bearing an electron-withdrawing group [32]. As with many ATR reactions, multi-dentate amines were critical for the reaction. These types of ligands in conjunction with copper catalysts are known to promote the formation of stabilized carbon-centered radicals [14]. In this reaction, the putative tertiary radical undergoes addition to a styrene derivative, creating a stabilized benzylic radical. Akin to classic ATR manifolds, the authors propose that this radical abstracts a bromine atom from copper, regenerating the copper(I) catalyst and forming a benzylic halide. Unlike classical ATR reactions, the resultant alkyl halide then undergoes E_2_ elimination, promoted by the included amine base, resulting in the observed product (Scheme 9). While the reaction conditions are similar to those utilized in atom transfer radical addition and polymerization reactions, the use of excess amine was critical for a robust alkenylation reaction and to avoid atom transfer and polymerization products.

**Scheme 10:**
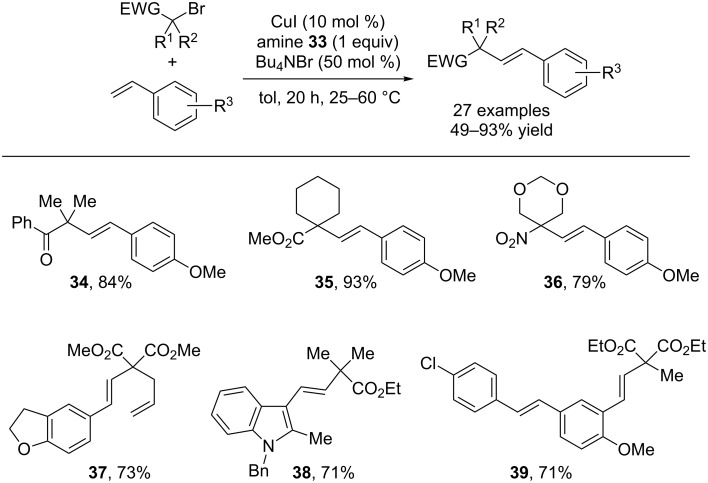
Scope of the copper-catalyzed alkenylation of tertiary electrophiles.

Various electron-withdrawing groups including esters, ketones and nitro groups were tolerated in the alkyl bromide coupling partner (Scheme 10). In addition, the reaction shows excellent functional group compatibility with respect to the styrene coupling partner, tolerating ethers, nitriles, chlorides and amines. Notably, internal alkenes (such as **39**) were not affected during the reaction. Although the reaction proceeds via a distinct mechanism, the authors refer to the transformation as a formal Heck-type alkenylation of alkyl bromides.

As with the nitroalkane alkylation chemistry illustrated above, this reaction is a rare example of a transition metal-catalyzed cross coupling of tertiary alkyl halides. These types of reactions are often very difficult because of the propensity of the intermediate tertiary alkylmetal to undergo β-hydrogen elimination [33–35]. By developing a reaction that proceeds via tertiary alkyl radicals, these strategies have elegantly circumvented this problem and have expanded the scope of alkyl cross-coupling reactions.

**Scheme 11:**
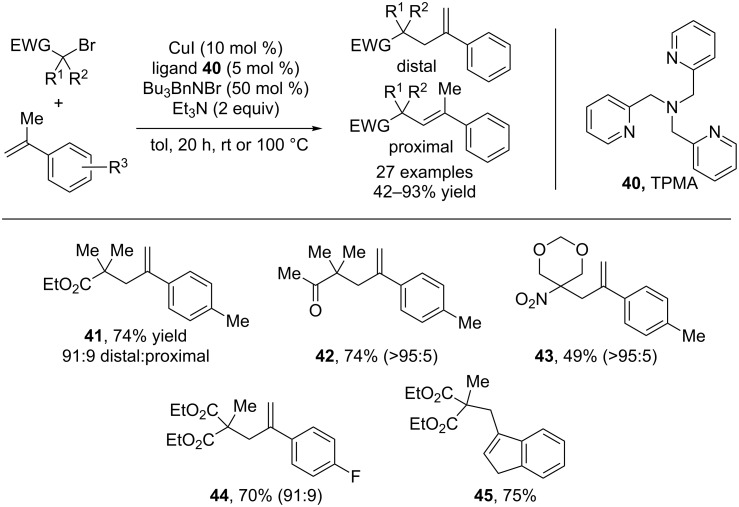
Scope of the exo-methylene styrene synthesis.

Nishikata has subsequently expanded the utility of this reaction manifold by elegant tailoring of the radical donor and acceptor [36]. If the styrene radical acceptor bears an alkyl group at the α-position, elimination happens preferentially from this position to provide exo-methylene styrenes (Scheme 11). In this case, tris(2-pyridylmethyl)amine (TPMA) was utilized as the ligand, along with excess amine base. Styrenes were produced in high yield with excellent selectivity for the exo-methylene products. The high levels of distal selectivity are proposed to arise from steric contributions. The intermediate alkyl bromide that results from the initial atom transfer possesses two possible sites of elimination. The authors propose that the distal hydrogen is more accessible to the bulky amine base. Similar to the aforementioned direct alkenylation, the reaction displays excellent functional group tolerance. α-Bromo esters, ketones, and nitroalkanes all coupled smoothly to form exo-methylene compounds. Again, excess amine was crucial to promote the desired transformation and supress polymerization.

**Scheme 12:**
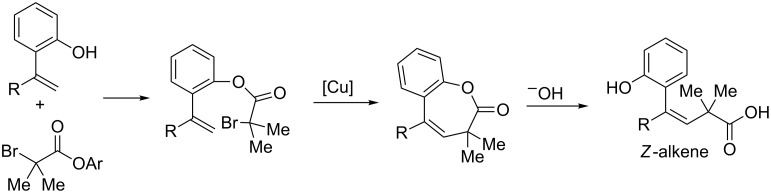
Phenol-directed synthesis of *Z*-alkenes.

**Scheme 13:**
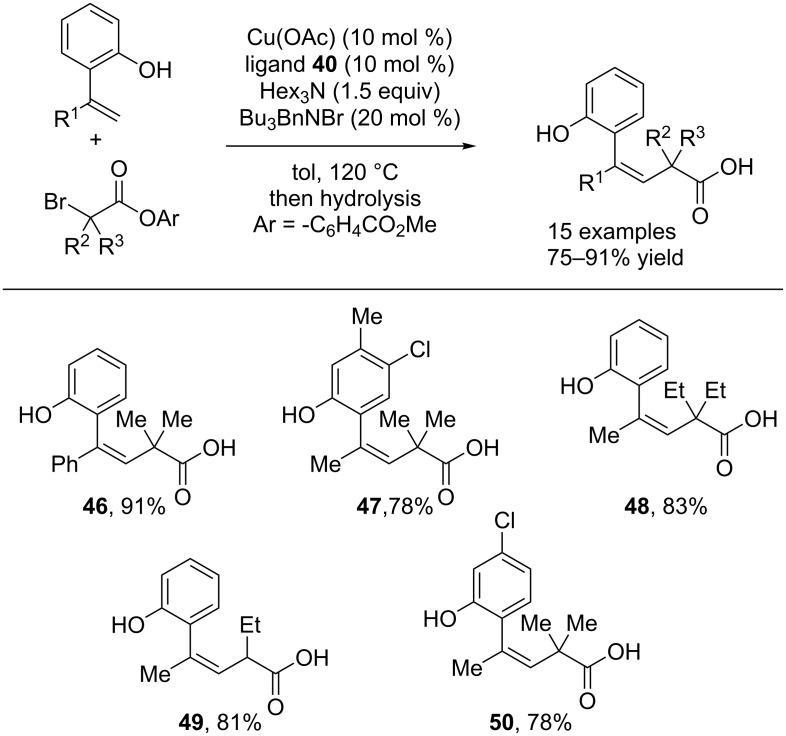
Scope of the phenol-directed *Z*-alkene synthesis.

A detachable ester group can be utilized in order to achieve *Z*-selective alkene synthesis [37]. In this protocol, an *ortho*-hydroxy substituted styrene undergoes esterification with a labile α-bromo ester. This positions the reactive radical exclusively on the face of the alkene leading to the *Z* product (Scheme 12). After cross coupling, the ester can be hydrolyzed to provide exclusively the *Z-*alkene product (Scheme 13). This protocol is complementary to the initially reported alkenylation of tertiary alkyl bromides, which resulted exclusively in *E*-alkene products.

**Scheme 14:**
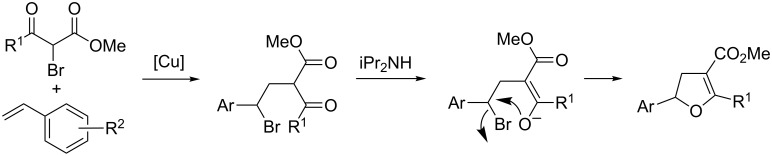
Rationale for the formal [3 + 2] cycloaddition.

**Scheme 15:**
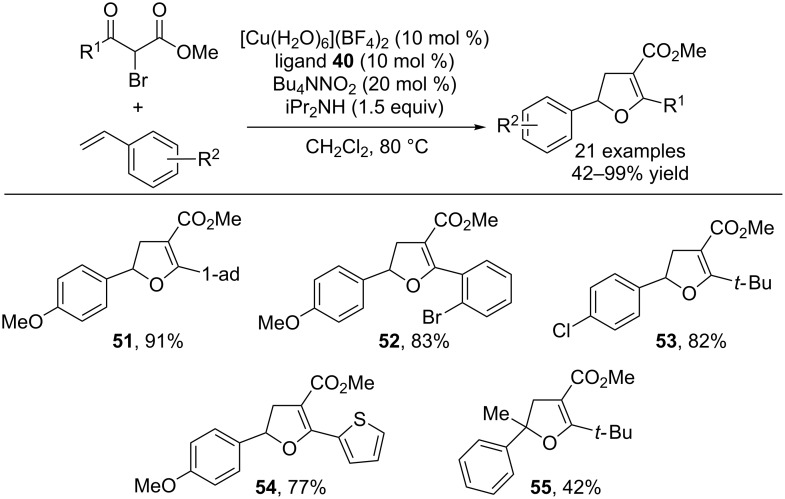
Scope of the formal [3 + 2] cycloaddition.

When the alkyl bromide coupling partner was changed to an α-bromo keto ester, the radical addition is followed by a tautomerization/cyclization event that provides dihydrofurans in excellent yields [38]. The radical formation/addition events proceed largely in the same manner as the aforementioned alkenylation reactions. However, the product of addition of a secondary bromo keto ester possesses an acidic α-proton. This proton can be deprotonated by diisopropylamine, and the resulting enolate cyclizes onto the newly formed benzylic halide to form the dihydrofuran (Scheme 14). This method is formally a [3 + 2] cycloaddition of styrenes and α-bromo keto esters. Using this method, various substituted dihydrofurans were produced in high yields (Scheme 15). Electron-rich and electron-poor styrenes as well as alkyl and aryl keto esters were combined to provide diversely substituted dihydrofurans. The dihydrofurans could be easily converted into furans using DDQ in a one-pot process.

**Scheme 16:**
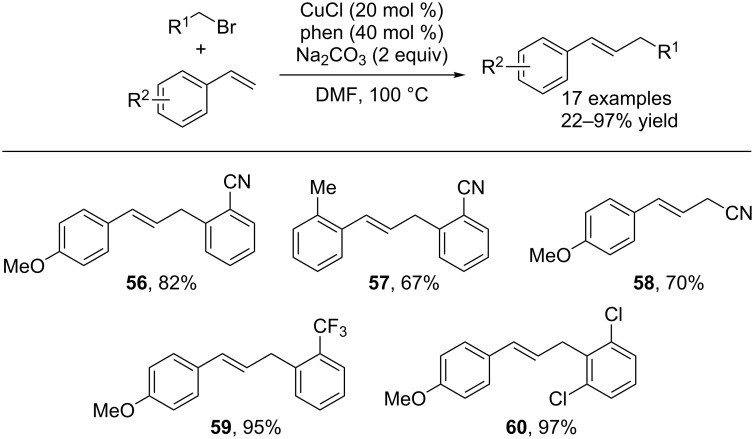
Benzylation of styrenes using copper catalysis.

In addition to α-bromo esters and ketones, Lei and co-workers have also recently shown that benzylic halides could undergo radical alkenylation via copper catalysis [39]. By exploiting the propensity of benzyl halides to form benzyl radicals under copper catalysis, an alkenylation similar to that developed by Nishikata was realized. Using a mixture of copper and 1,10-phenanthroline, benzylic halides were directly alkenylated using styrenes as coupling partners. Under these conditions, various allylbenzene derivatives were synthesized in very good yields (Scheme 16). Interestingly, the CuCl/phenanthroline catalyst system was also capable of cross coupling styrenes with α-bromocarbonyls and α-bromonitriles. EPR studies confirmed the presence of radical intermediates.

### Additions to alkynes

**Scheme 17:**
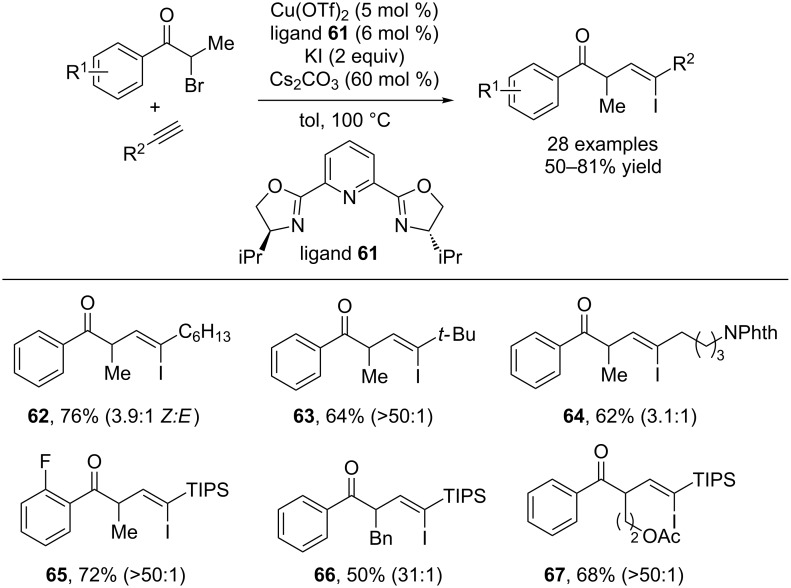
Copper-catalyzed carboiodination of alkynes.

Recently, ATR type catalysts have also been used to catalyze the addition of alkyl radicals to alkynes, with reincorporation of the halide to form substituted vinyl halides. Hu and co-workers reported that a copper/PyBox catalyst system could catalyze the addition of phenacyl iodides, generated in situ from the corresponding bromides and potassium iodide, across alkynes to provide substituted vinyl iodides (Scheme 17) [40]. The process selectively forms the *Z*-alkenes. Although the *E/Z* ratios are often modest, the products are highly useful synthetic building blocks that may be used in subsequent cross-coupling reactions. The mechanism is proposed to proceed similarly to the alkenylation reactions described above. The α-iodoketone is proposed to form an alkyl radical, which adds to the alkyne producing an sp^2^ centered radical which abstracts an iodine atom from another α-iodoketone to restart the catalytic cycle.

**Scheme 18:**
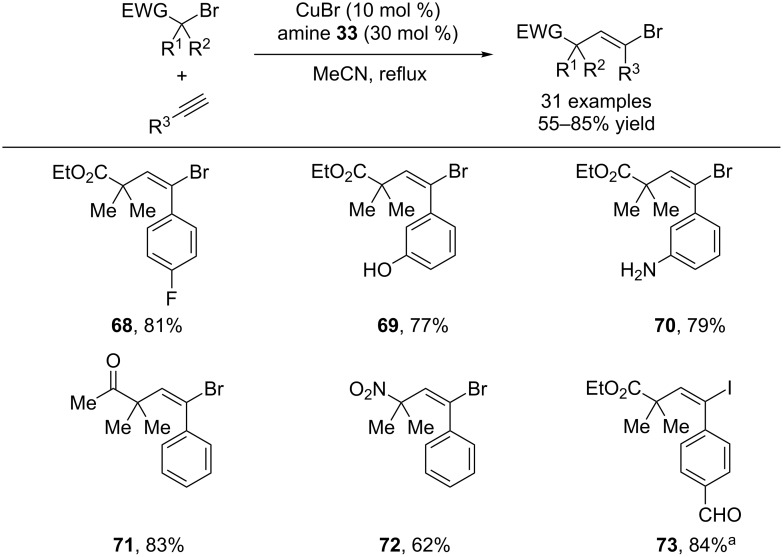
Copper-catalyzed *trans*-carbohalogenation of alkynes. ^a^NaI (2 equiv) was added.

In a complementary protocol, Zhu and co-workers have discovered a selective *trans*-carbohalogenation of aryl alkynes [41]. A tridentate amine was used as a ligand for the copper catalyst. Using these conditions, highly valuable vinyl bromides were synthesized in excellent yields. Various functionalized tertiary alkyl bromides participated in the reaction, including those bearing esters, ketones, nitro and nitriles (Scheme 18). If the reaction was run in the presence of sodium iodide, vinyl iodides were produced. This protocol is complementary to Hu’s work in that it provides products of *trans*-carbohalogenation. In an elegant one-pot procedure, enynes could be synthesized by introducing a second alkyne and a palladium catalyst to perform a tandem carbohalogenation/Sonagashira coupling.

## Conclusion

Copper catalysis has recently emerged as a new means of harnessing the potential of alkyl radicals in catalytic alkylation chemistry. While the groundwork for this recent surge was laid in the development of atom-transfer radical addition and polymerization chemistry, the realization of its potential in alkylation chemistry will greatly expand the use of copper catalysis as a means for constructing complex molecular architectures. The recent groundswell in copper-catalyzed radical additions is likely to continue, with new and creative methods being developed to take advantage of the unique selectivity and reactivity observed in this class of reaction.
